# Burnout among German oncologists: a cross-sectional study in cooperation with the *Arbeitsgemeinschaft Internistische Onkologie* Quality of Life Working Group

**DOI:** 10.1007/s00432-022-03937-y

**Published:** 2022-02-13

**Authors:** Madeleine Helaß, Georg Martin Haag, Ulli Simone Bankstahl, Deniz Gencer, Imad Maatouk

**Affiliations:** 1 Department of General Internal Medicine and Psychosomatics, Heidelberg University Hospital, Heidelberg, Germany; 2 Department of Medical Oncology, National Centre for Tumour Diseases (NCT), Heidelberg University Hospital, Heidelberg, Germany; 3 Institute for Clinical-Oncological Research (IKF), Krankenhaus Nordwest gGmbH, Frankfurt, Germany; 4 Department of Medicine III, Medical Faculty Mannheim, University Medical Center Mannheim, Heidelberg University, Heidelberg, Germany; 5 Section of Psychosomatic Medicine, Psychotherapy and Psycho-Oncology, Department of Internal Medicine II, Julius-Maximilian University Würzburg, Würzburg, Germany; 6 Psychosomatics, Psychotherapy and Psychooncology, University Hospital Würzburg, Medical Clinic II, Oberdürrbacher Str. 6, 97080 Würzburg, Germany

**Keywords:** Burnout, Exhaustion, Disengagement, Oldenburg burnout inventory, Oncologist, Prevalence

## Abstract

**Purpose:**

Oncologists are at an increased risk of developing burnout, leading to negative consequences in patient care and in professional satisfaction and quality of life. This study was designed to investigate exhaustion and disengagement among German oncologists and assess the prevalence of burnout among oncologists within different professional settings. Furthermore, we wanted to examine possible relations between sociodemographic factors, the oncological setting, professional experience and different aspects of burnout.

**Methods:**

In a cross-sectional study design, an Internet-based survey was conducted with 121 oncologists between April and July 2020 using the Oldenburg Burnout Inventory, which contains items on exhaustion, disengagement, and burnout. Furthermore, sociodemographic data of the participants were assessed. The participants were members of the Working Group Medical Oncology (*Arbeitsgemeinschaft Internistische Onkologie*) within the German Cancer Society.

**Results:**

The survey showed a burnout prevalence of 43.8%, which correlated with age and professional experience; that is, the prevalence is particularly high among younger oncologists. Exhaustion is closely related to employment status; that is, it was significantly higher among employed oncologists. There were remarkably low levels of disengagement among oncologists, highlighting the own demand to fulfil job requirements despite imminent or actual overburdening in daily work.

**Conclusion:**

More support is necessary to mitigate the professional stressors in the healthcare system. To ensure quality medical care, employees should be offered preventive mental health services early in their careers.

## Introduction

Since the second half of the last century, burnout among healthcare workers in hospitals has been becoming the focus of health psychology and occupational medicine research. Compared with the general population, health care professionals are at an increased risk of developing symptoms of burnout (Shanafelt et al. 2012; Dyrbye et al. 2014). Maslach’s fundamental work defined burnout as work-related stress in terms of three components: emotional exhaustion (EE), depersonalization (DP) and low personal accomplishment (PA). Moreover, to date, the Maslach Burnout Inventory (MBI) (Maslach et al. 1981) is the most widely used and accepted survey to assess burnout (Rotenstein et al. 2018).

During the last 20 years, an increasing prevalence of burnout among physicians has been observed (Linzer et al. 2001; Eelen et al. 2014; Shanafelt et al. 2015), though the prevalence rates vary significantly between different surveys depending on the analyzed diagnostic criteria, demographic factors and medical discipline (Prins et al. 2007; Yates & Samuel 2019). The highest rates were observed in emergency care, neurology and general internal medicine (Shanafelt et al. 2012, 2015), especially among oncologists (Yates and Samuel 2019).

Several factors have been identified as risk factors for physicians developing burnout, including workload (Cooper et al. 1989; Freeborn 2001). Among these factors are inefficient work processes (Shanafelt, et al. 2016a, b), time pressure (Kleiner and Wallace 2017) and administrational issues (Sinsky et al. 2016). Beyond that, oncologists are often confronted with patients deteriorating significantly or dying due to an advancing tumor disease, despite their efforts in terms of oncological care (Trufelli et al. 2008), focusing not only on prolongation of survival but also on improving or maintaining the patient's quality of life. Thus, apart from tumor-specific therapy, concomitant supportive treatment is integral part of oncologist's daily work to reach this goal.

Burnout among physicians can have devastating effects on patient care, including a significantly increased risk of medical errors (Shanafelt et al. 2010; Hall et al. 2016), a decreased job productivity (Dewa et al. 2014; Shanafelt et al. 2016) and a lower patient satisfaction rate (Haas et al. 2000; Halbesleben and Rathert 2008). Furthermore, physicians with burnout may suffer from somatic and mental health problems, including depression and substance abuse (Oreskovich et al. 2012; Medisauskaite and Kamau 2017), an increased risk of vehicular accidents (West et al. 2012) and an increased suicide risk (van der Heijden et al. 2008). Hence, the assessment of the prevalence of burnout among clinically active oncologists is of great interest to researchers, clinicians, and policy makers.

The prevalence of burnout repeatedly leads to debates (Banerjee et al. 2017; Bianchi et al. 2017), and the MBI (Maslach et al. 1981) has revealed some psychometric weaknesses (i.e. factor validity and one-sided wording of items) (for further information, see Demerouti et al. 2003; Demerouti and Bakker 2008). A verification of the prevalence via repetitive measurements of the burnout diagnostic criteria seems to be necessary. Therefore, this study was designed to investigate two dimensions of burnout using a well-validated questionnaire in a German sample of oncologists working in different oncological settings. Furthermore, we wanted to examine possible relations between sociodemographic factors, the oncological setting, and work experience and different aspects of burnout.

## Materials and methods

### Study population

All members of the Working Group Medical Oncology (*Arbeitsgemeinschaft Internistische Onkologie (AIO)*) within the German Cancer Society were asked via email to participate in an Internet-based survey. The inclusion criteria were a minimum age of 18 years and a medical practice with patients with cancer. This cross-sectional study, platform provided by SoSci Survey, was open for 3 months from April to July in 2020. The access was only possible using a hyperlink sent in an email circular sent to all AIO members. Participation in the survey was voluntary and anonymous. The manuscript was prepared according to the Strengthening the Reporting of Observational Studies in Epidemiology statement criteria (Von Elm et al. 2007).

### Questionnaire

The 16-item Oldenburg Burnout Inventory (OLBI) (Demerouti and Bakker 2008) was assessed to measure the two following subscales—*exhaustion* and *disengagement* (8 items per subscale)—on a 5-point Likert scale (1 = “strongly disagree” to 5 = “strongly agree”). Four items of each subscale were inverted. The exhaustion dimension (OLBI_exh_) refers to the “general feelings of emptiness, overtaxing from work, a strong need for rest and a state of physical exhaustion” (Demerouti and Bakker 2008, p. 10). Exhaustion refers to the long-term consequence of intense physical, affective, and cognitive stress, while emotional exhaustion (MBI_EE_) surveys only affective aspects. Depersonalization (MBI_DP_) refers to distancing from a beneficiary and is only one aspect of the disengagement scale (OLBI_dis_). OLBI_dis_ refers to “distancing oneself from one’s work and the associated attitudes and attitudes toward work, to negative cynical attitudes and behaviors toward one’s work in general” (ibid., p. 10). The last issue in particular seemed crucial, as work-related attitudes are predictors of job performance (Hettiararchchi and Jayarathna 2014), and job involvement is related to job satisfaction (Awadh and Wan Ismail 2012).

Both subscales had a reliability of 0.85. Bivariate correlations between both subscales were 0.55 (*p* < 0.01) for healthcare workers with mean levels of exhaustion (*M* = 2.53) and disengagement (*M* = 2.38) (Demerouti and Bakker 2008). Since there is no standard cutoff for burnout in the OLBI, we first used M_OLBI_ ≥ 2.18 as the mean value of the disengagement and exhaustion scales (Peter Chernoff et al. 2019). Further analyses are limited to the more sensitive cutoff of M_Exh_ ≥ 2.5 (Block et al. 2020) for the exhaustion subscale as a “burnout measure.”

Furthermore, sociodemographic data, profession, medical field, professional experience, workplace, and employment status, board certification, work on inpatient palliative care unit (PCU) and participation in an outpatient palliative care team (*Spezialisierte Ambulante Palliativversorgung* (SAPV)) were assessed, including the estimated proportion of total working time, proportion of inpatient work, working hours with tumor patients and the estimated proportion of working time with palliative patients.

### Statistics

To evaluate exhaustion, disengagement, and burnout among German oncologists, absolute and relative frequencies for categorical data, mean values, standard deviation, and range for continuous variables were calculated. Participants whose data were incomplete were nevertheless included, as the missing data only included some sociodemographic data. First, we used one-way analysis of variance (ANOVA) as an alternative to *t* test to examine differences in the subgroups of sociodemographic variables in exhaustion, disengagement, and burnout. Second, correlation coefficients were measured among all study variables to examine possible relationships between them. Third, multiple regression analyses were performed between disengagement and exhaustion as dependent variables and the sociodemographic factors as independent variables to examine possible predictors for the OLBI measures. The risk ratio (RR) was used to determine the risk of burnout. For all tests, p-values of less than 0.05 were used to indicate statistical significance. Analyses were done using Statistical Package for the Social Sciences, version 27 (IBM, 2020).

Informed consent was obtained from all participants. Ethical Approval was received on September 2019 from the Ethical Committee of the Medical Faculty in Heidelberg (S-615/2019). The study was registered to the German Clinical Trials Register (DRKS500018851).

## Results

### Characteristics of participants

Approximately 1400 physicians were contacted, among whom 121 participated, which corresponds to a response rate of 8.64%. The mean age was 50.28 ± 9.32 years (range, 28–75 years). This corresponds to the distribution of all age groups of AIO members (40–60 years, 49.14%) and represents the biggest age cohort of physicians, board certified in internal medicine in Germany (40–60 years, 59.8%) (Bundesärztekammer 2020). The sample was predominantly male (*n* = 80, 66%), reflecting the majority of AIO members (male 77, 59%) as well as the majority of German oncologists (male 59.66%). Professional experience varied widely between 2 and 49 years (median = 20–24 years (26%, interquartile range (IQR) = 15–29 years). Moreover, 113 (94.9%) physicians had a board certification in hemato-oncology. The participants worked on average 82.40% ± 17.13% (range, 21%–100%) of their time with patients with tumor. Twenty-one (17.4%) physicians worked within a PCU, spending 27.81% ± 24.11% (range 6–81%) of their working time, whereas 11 (9.1%) physicians participated in an outpatient palliative care team (*SAPV*), accounting for 11.64% ± 8.51% (range 2–34%) of their working time. Absolute and relative frequencies are shown in Table 1. Table 2 presents the mean values, standard deviations, ranges, and correlation coefficients. Risk ratios are shown in Table 3.

**Table 1:** Descriptive statistics of sociodemographic factors in disengagement and exhaustion

	Disengagement						Exhaustion					
	Low^1^		Medium		High		Low		High (burnout)^2^		Total	
	*n*	%	*n*	%	*n*	%	*n*	%	*n*	%	*n*	%
	60	49.6	46	40.5	15	12.4	68	56.2	53	43.8	121	100
Gender												
Male	37	30.6	31	29.8	12	9.9	45	37.2	35	28.9	80	66.1
Female	23	69.4	16	13.2	3	2.5	23	19.0	18	14.9	41	33.9
Status of Employment												
Self-employed in oncological practice Individual practice	2	1.7	–	–	1	0.8	2	1.7	1	0.8	3	2.5
Group practice	8	6.6	13	10.7	3	2.5	16	13.2	8	6.6	24	19.8
Employed In oncological practice/medical care centre	7	5.8	3	2.5	1	0.8	5	4.1	6	5.0	11	9.1
In hospital	41	33.9	29	24.0	10	8.3	42	34.7	38	31.4	80	66.1
Not specified	2	1.7	1	0.8	–	–	3	2.5	–	–	3	2.5
Medical field												
Internal medicine Hematology/oncology	47	38.8	39	32.2	13	10.7	56	46.3	43	35.5	99	81.8
Gastroenterology	1	0.8	4	3.3	1	0.8	1	0.8	4	3.3	5	4.1
Pneumology	2	1.7	1	0.8	–	–	2	1.7	1	0.8	3	2.5
Gynecology	2	1.7	1	0.8	–	–	2	1.7	1	0.8	3	2.5
Radiation therapy	2	1.7	–	–	–	–	1	0.8	1	0.8	2	1.7
Neurology	–	–	–	–	–	–	–	–	–	–	–	–
Surgery	–	–	1	0.8	1	0.8	–	–	2	1.7	2	1.7
Other	5	4.1	1	0.8	–	–	5	4.1	1	0.8	6	5
Not specified	1	0.8	–	–	–	–	1	0.8	–	–	1	0.8
Type of specialists												
Internal medicine	2	1.7	1	0.8	–	–	2	1.7	1	0.8	3	2.5
Hematology/oncology	43	35.5	34	28.1	10	8.3	53	43.8	34	28.1	87	71.9
Gastroenterology	1	0.8	3	2.5	1	0.8	-	-	5	4.1	5	4.1
Pneumology	2	1.7	1	0.8	–	–	2	1.7	1	0.8	3	2.5
Radiation therapy	2	1.7	1	0.8	–	–	1	0.8	2	1.7	3	2.5
No specialist	5	4.1	4	3.3	3	2.5	11	9.1	1	0.8	12	9.9
Another specialist	5	4.1	2	1.7	1	0.8	4	3.3	4	3.3	8	6.1
PCU^3^												
Yes	13	10.7	7	5.8	1	0.8	14	11.6	7	5.8	21	17.4
No	45	37.2	38	31.4	14	11.8	52	43.0	45	37.2	97	80.1
Not specified	2	1.7	1	0.8	–	–	2	1.7	1	0.8	3	2.5
SAPV^4^												
Yes	8	6.6	7	5.8	2	1.7	9	7.4	2	1.7	11	9.1
No	50	41.3	43	35.5	13	10.7	56	46.3	50	41.3	106	88.4
Not specified	2	1.7	2	1.7	–	–	3	2.5	1	0.8	4	2.5
Professional experience												
< 2	–	–	–	–	–	–	–	–	–	–	–	–
2–4	3	2.5	2	1.7	3	2.5	3	2.5	5	4.1	8	6.6
5–9	4	3.3	3	2.5	2	1.7	5	4.1	4	3.3	9	7.4
10–14	4	3.3	7	5.8	–	–	4	3.3	7	5.8	11	9.1
15–19	9	7.4	6	5.0	2	1.7	8	6.6	9	7.4	17	14
20–24	14	11.6	10	8.3	2	1.7	13	10.7	13	10.7	26	21.5
25–29	15	12.4	2	1.7	2	1.7	15	12.4	4	3.3	19	15.7
30–34	6	5.0	10	8.3	2	1.7	11	9.1	7	5.8	18	14.9
35–39	1	0.8	7	5.8	2	1.7	4	3.3	4	3.3	8	6.6
40–44	1	0.8	–	–	2	1.7	1	0.8	–	–	1	0.8
> 45	1	0.8	–	–	–	–	1	0.8	–	–	1	0.8
Not specified	2	1.7	1	–	–	–	2	1.7	–	–	3	2.5

^1^Low (< 1.63), medium (1.64–2.24) and high (> 2.24)

^2^Cutoff = 2.5

^3^PCU = Palliative Care Unit Activity

^4^SAPV = Spezialisierte Ambulante Palliativversorgung (outpatient palliative care)

**Table 2:** Mean, standard deviations, ranges, and correlation coefficients

No.					1	2	3	4	5	6	7	8	9	10	11	12	13	14	15	16	17
	Item	M	SD	Range																	
1	Age	50.28	9.32	28–75	1																
2	Gender				0.253^**^	1															
3	Professional experience				0.830**	0.281	1														
4	Status of employment				− 0.401**	0.189	0.452***	1													
5	Board certification				0.295**	0.331*	0.411***	0.232	1												
6	Medical field				0.148	0.244	0.256	0.212	0.721**	1											
7	Work time with patients with tumor %	82.40	17.13	21–100	** − 0**.100	** − 0**.107	** − 0**.066	** − 0**.004	** − 0**.151	** − **0.118	1										
8	Palliative patients with tumor %	60.81	21.02	9–98	** − 0**.060	0.030	** − 0**.018	0.089	− 0.373^**^	− 0.248^**^	0.222^*^	1									
9	Inpatient work %	41.56	39.95	0–100	− 0.286**	** − 0**.012	− 0.194**	0.660***	** − 0**.048	0.125	0.000	** − **0.116	1								
10	Working on a palliative care unit				** − 0**.048	0.039	**0.**291	0.262*	0.291	0.265	** − **0.128	− 0.227^*^	− 0.265^**^	1							
11	Palliative care %	27.81	24.11	6–81	0.263	0.435^*^	0.232	0.367	** − **0.083	0.651^**^	0.037	0.442^*^	0.446^*^	-	1						
12	SAPV				** − 0**.082	0.015	**0.**192	0.265^*^	0.192	0.362^*^	** − **0.109	** − **0.159	0.121	0.163	** − 0**.421	1					
13	SAPV%	11.64	8.51	2–34	0.007	0.362	** − 0**.143	** − 0**.329	** − **0.235	** − **0.200	− 0.755^**^	0.011	** − **0.307	0.246	0.758	-	1				
14	Exhaustion	2.33	0.60	1.13–3.75	− 0.183^*^	0.053	− 0.180**	0.232^*^	0.033	0.072	** − 0**.077	0.064	0.170	0.075	0.007	0.138	0.607^*^	1			
15	Disengagement	1.65	0.45	1.00–2.88	** − 0**.024	0.153	0.037	** − 0**.078	** − **0.099	** − **0.034	** − **0.040	0.138	0.020	0.102	0.098	0.027	0.097	0.533*^**^	1		
16	Exhaustion > 2.5 (burnout)				** − 0**.119	0.008	0.179	0.114	0.257	0.251	** − **0.093	0.020	0.105	0.167	0.138	0.180	0.871***	0.794^***^	0.591^***^	1	
17	Disengagement > 2.24				** − 0**.050	0.166	** − 0**.047	0.172	0.172	0.228	0.170	0.088	0.000	0.151	− 0.097	− 0.097	− 0.240	0.253^**^	0.656^***^	0.313^**^	1

*M* mean, *SD* standard deviation, *SAPV* Spezialisierte Ambulante Palliativversorgung (outpatient palliative care)

Significant correlations are printed in bold

*Correlation is significant at a level of *p* < 0.05(2-tailed)

**Correlation is significant at a level of *p* < 0.01 (2-tailed)

***Correlation is significant at a level of *p* < 0.001(2-tailed)

**Table 3:** Relative ratio for burnout among German oncologists

	Cutoff/ Categories	Value	*N* = 53	%	RR [95% CI]	Pearson -Chi	*p*^1^
Age (years)	P25 = 43	< 43	14	50.0	1.192 [0.767–1.853]	0.569	0.451
		> 43	39	41.9	0.839 [0.540–1.303]		
	P50 = 52	** < 52**	**33**	**55.0**	**1.618 [1.096–2.568]**	**6.063**	**0.014**
		** > 52**	**20**	**32.8**	**0.596 [0.389–0.913]**		
	P75 = 57	< 57	42	46.7	1.315 [0.779–2.220]	1.171	0.278
		> 57	11	35.5	0.760 [0.450–1.283]		
Work time with patients with tumor (%)	P25 = 76	< 76	15	55.6	1.330 [0.878–2.016]	1.602	0.206
		≥ 76	38	41.8	0.752 [0.496–1.139]		
	P50 = 86	< 86	29	51.8	1.338 [0.895–2.001]	2.033	0.154
		≥ 86	24	38.7	0.747 [0.500–1.118]		
	P75 = 96	< 96	41	47.7	1.271 [0.772–2.094]	0.976	0.323
		≥ 96	12	37.5	0.787[0.478–1.290]		
Palliative patients with tumor (%)	P25 = 48	< 48	11	39.3	0.842 [0.565–1.403]	0.470	0.493
		≥ 48	42	46.7	1.188 [0.713–1.980]		
	P50 = 65	< 65	26	44.8	0.996 [0.668–1.486]	0.000	0.985
		≥ 65	27	45.0	1.004 [0.673–1.497]		
	P75 = 78.25	< 78.25	41	46.1	1.113 [0.683–1.814]	0.194	0.659
		≥ 78.25	12	41.4	0.898 [0.551–1.463]		
Inpatients (%)	P25 = 6	< 6	13	52.0	1.183 [0.760–1.841]	0.511	0.475
		≥ 6	40	44.0	0.845 [0.543–1.313]		
	P50 = 34,5	< 34.5	26	44.8	0.963 [0.647–1.432]	0.035	0.852
		≥ 34.5	27	46.6	1.038 [0.698–1.544]		
	P75 = 76.75	< 76.75	39	44.8	0.929 [0.596–1.446}	0.104	0.747
		≥ 76.75	14	48.3	1.077 [0.691–1.677]		
PCU^2^ (%)	P25 = 7	< 7	2	40.0	1.280 [0.350–4.680]	0.131	0.717
		≥ 7	5	31.3	0.781 [0.214–2.850]		
	P50 = 18	< 18	5	50.0	2.750 [0.679–11.134]	2.386	0.122
		≥ 18	2	18.2	0.364 [0.090–1.472]		
	P75 = 43	< 43	5	31.3	0.787 [0.214–2.856]	0.131	0.717
		≥ 43	2	40.0	1.280 [0.350–4.680]		
SAPV^3^ (%)	P25 = 6	< 6	1	50.0	4.500 [0.447–45.328]	1.664	0.197
		≥ 6	1	11.0	0.222 [0.022–2.238]		
	P50 = 11	< 11	1	20.0	0.835 [0.068–10.20]	0.020	0.887
		≥ 11	1	16.7	1.200 [0.098–14.69]		
	P75 = 12	< 12	1	12.5	0.375[0.033–4.275]	0.637	0.425
		≥ 12	1	33.3	2.667 [0.234–30.399]		
Professional experience (years)	P25 = 15–19	< 15	16	57.1	1.390 [0.927–2.084]	2.218	0.139
		≥ 15	37	41.1	0.719 [0.480–1.079]		
	P50 = 20–24	< 20	25	55.6	1.448 [0.9800–2.141]	3.329	0.068
		≥ 20	28	38.4	0.690 [0.467–1.021]		
	**P75 = 25–29**	** < 25**	**38**	**53.5**	**1.677 [1.048–2.684]**	**5.336**	**0.021**
		** ≥ 25**	**15**	**31.9**	**0.596 [0.373–0.955]**		
Gender	Male		35	43.9	0.997 [0.715–1.391]	0.000	0.987
	Female		18	43.8	1.003 [0.655–1.536]		
PCU	Yes		7	33.6	7.11 [0.372–1.350]	1.280	0.258
	No		45	46.9	1.255 [0.879–1.372]		
SAPV	Yes		2	18.2	0.385 [0.108–1.372]	3.392	0.660
	No		50	47.2	1.549 [1.112–2.158]		
Status of employment	Self-employed		9	33.3	0.613 [0.301–1.251]	1.898	0.168
	Employed		44	48.8	1.148 [0.949–1.393]		
Board certification	Yes		47	43.1	0.973 [0.861–1.089]	0.208	0.648
	No		6	50.0	1.283 [0.439–3.752]		
Board certification internal medicine: hematology/oncology	Yes		34	39.1	0.823 [0.649–1.044]	2.804	0.094
	No		19	55.9	1.635 [0.916–2.885]		
Medical field: Internal medicine: Hematology/oncology	Yes		43	43.4	0.971[.821–1.148	0.123	0.726
	No		10	47.6	1.149 [.528–2.499]		

Statistically significant differences are printed in bold

^1^Significant at a level of *p* < 0.05 (2-tailed)

^2^PCU = Palliative Care Unit Activity

^3^SAPV = Spezialisierte Ambulante Palliativversorgung (outpatient palliative care)

### Disengagement

**Fig. 1:**
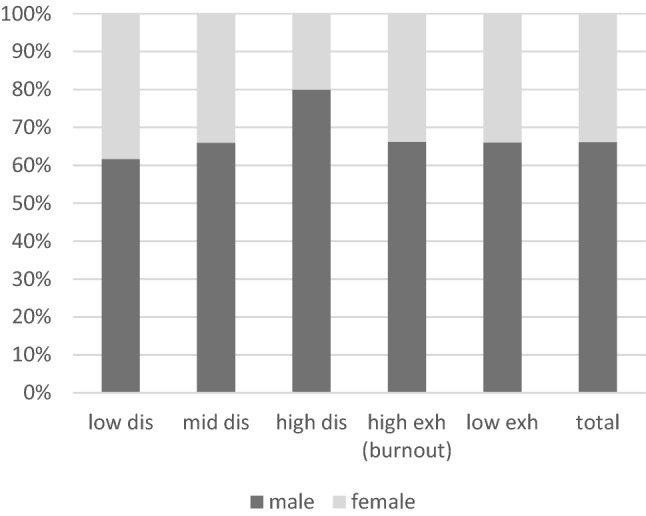
Gender distribution of disengagement (i.e. low, middle and high) and exhaustion (i.e. low and high [burnout])

The mean value on the disengagement scale was 1.65 ± 0.45 (range, 1.00–2.88). To evaluate the disengagement scale, the range of values was divided into three parts and designated as low (< 1.63), medium (1.63–2.24) and high (> 2.24). Moreover, 60 (49.59%) physicians showed low disengagement (mean = 1.28 ± 0.153; range = 1–1.5), 46 (38.02%) showed medium disengagement (mean = 1.85 ± 0.19; range 1.63–2.13) and 15 (12.40%) showed high disengagement (mean = 2.49 ± 0.21; range 2.38–2.88). High disengagement was mainly related to men (*n* = 12, 9.9%) and hospital employees (*n* = 10, 8.3%) (Fig. 1).

Correlation analyses, ANOVA, and regression analyses showed no significant correlations between the disengagement scale and other variables.

### Exhaustion

Based on a cutoff of M_OLBI_ = 2.18, 45 physicians (52.3%) of the total sample had burnout. Focusing on the more specific exhaustion scale with a cutoff of M_Exh_ > 2.5, 53 (43.8%) physicians showed increased values in the exhaustion scale as a hint for suffering from burnout.

Pearson’s correlation coefficients showed a significant negative correlation between age and exhaustion (*r* =  − 0.183, *p* < 0.01); that is, the older the physician, the less exhaustion. Using a cutoff at the highest quartile (P75), physicians older than 57 years (*n* = 31) have significantly lower values (mean = 2.09 ± 0.94) on the exhaustion scale than physicians younger than 57 years (*n* = 90; mean = 2.41 ± 0.57) (*t*(119) =  − 2,581; *p* < 0.05).

**Fig. 2:**
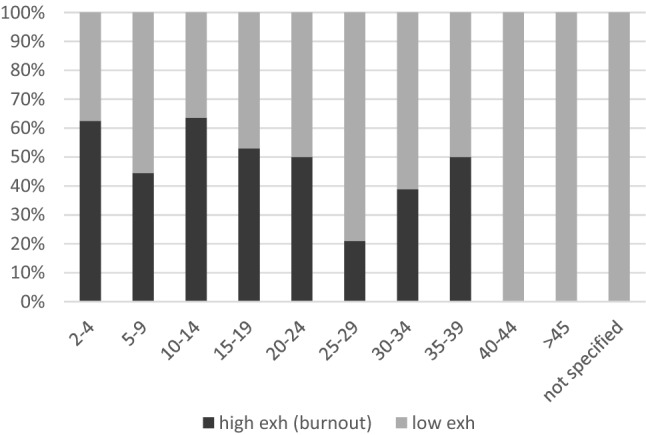
Professional experience and exhaustion (burnout)

A significantly strong positive correlation was observed between age and professional experience (*r* = 0.830; *p* < 001). Kendall’s Tau showed a significant negative correlation between professional experience and exhaustion (*r* =  − 0.180; *p* < 0.01); that is, the less work experience, the greater the exhaustion. Pearson’s chi-square test confirmed an association between professional experience of less than 20 years with burnout (M_Exh_ ≥ 2.5): χ^2^(1) = 5.176; *p* < 0.05, *φ* = 0.209. The risk of burnout among physicians with a professional experience of less than 25 years (*n* = 38, 53.5%) was thrice higher than that among physicians with a professional experience of at least 25 years (*n* = 25, 31.9%) (RR = 1.677, 95% CI 1.129–6.419; *p* < 0.05) (Fig. 2).

Pearson’s correlation coefficients showed a slightly positive correlation between employment status and exhaustion (*r* = 0.232; *p* < 0.05). The difference in exhaustion between employed (mean = 2.415 ± 0.582) and self-employed (mean = 2.08 ± 0.60) physicians was significant (*t*(119) = 2.576; *p* < 0.05), suggesting that employed physicians describe themselves as more exhausted than self-employed colleagues. The comparison of the mean values of exhaustion showed a significant difference (*t*(114) = 2.115; *p* < 0.05) between physicians treating less than 30% of inpatients (*n* = 55; mean = 2.22 ± 0.593) and physicians treating more than 30% of inpatients (mean = 2.45 ± 0.609). Moreover, 35 (32.4%) employed and seven (9.6%) self-employed physicians showed signs of burnout; the relative risk was insignificant.

Physicians participating in an outpatient palliative care team (SAPV) were predominantly male (*n* = 7, 63.6%) with a mean age of 52.82 ± 5.741 years (range 41–60 years) and a professional experience between 5 and 34 years (median = 20–24 years [27.3%, IQR = 15–29 years]). They were predominantly hemato-oncology specialists (*n* = 9, 81.8%), self-employed in oncological joint practice (*n* = 5, 45%) or employed in a hospital (*n* = 5, 45%) and predominantly did not work in an inpatient PCU (*n* = 7, 63.6%). Physicians additionally working in inpatient palliative care settings (*n* = 4) on average worked 48.25% ± 34.40% (range 4–81%) of their time on PCUs.

Pearson’s correlation coefficients were calculated to evaluate the correlation between exhaustion and the percentage of engagement in an outpatient palliative care team (SAPV). The results showed a strongly positive correlation (*r* = 0.607; *p* < 0.05); that is, the higher the percentage of working time in SAPV was, the higher the level of exhaustion was.

## Discussion

In this study, we assessed the prevalence of burnout among oncologists and its possible relation to different sociodemographic factors. Using stringent diagnostic criteria according to the OLBI, more than 43.8% of participating German oncologists showed increased exhaustion rates as a sign of burnout. Though this prevalence is lower than the rate of burnout-affected physicians in other disciplines, such as emergency medicine, general internal medicine and neurology (Shanafelt et al. 2012; Peter Chernoff et al. 2019), our data reflect a public health crisis with a huge negative impact on patient care, physicians’ health and healthcare organizations and systems (reviewed by West et al. 2018).

The analysis of different healthcare systems showed broad variations regarding the prevalence of burnout among oncologists: Using different diagnostic criteria, even higher rates of burnout were reported in a survey among oncologists in the US (Allegra et al. 2005) or Korea (Lee et al. 2020), whereas a recent survey involving Italian oncologists has only shown a burnout rate of 10.5% (Cheli et al. 2021).

In this survey, burnout was significantly associated with age and professional experience, with a higher prevalence observed among younger physicians. These data are in line with published results emphasizing the higher risk of burnout among younger physicians at a lower hierarchical level (e.g. residents) (Shanafelt et al. 2012). In contrast, a higher age was associated with a lower prevalence of burnout in this survey, as shown in other surveys involving oncologists in different countries (Alacacioglu et al. 2009; Shanafelt et al. 2014). Higher weekly working hours, higher rates of emotional labor and more alternating shifts, including night or weekend work, might contribute to this increased prevalence (Panagopoulou et al. 2006). Furthermore, working in an inpatient setting and being confronted daily with severely sick patients in an advanced stage of disease might be additional cofactors. Furthermore, the double burden of managing family life and professional career can cause role conflicts as an additional risk factor for burnout (Linzer et al. 2001; Cheli et al. 2021). Interventions to promote the mental health of oncology workers should address these issues. Several authors (Blanchard et al. 2010; Roth et al. 2011; Shanafelt et al. 2014; Cheli et al. 2021) have proposed a higher prevalence of burnout in female physicians, whereas we and other groups (Wang et al. 2014) could not confirm this observation. However, the number of female physicians participating in this survey was low to draw any conclusions on gender-specific differences.

The decrease in the rate of burnout with advanced age and professional experience probably reflects the capability of physicians to deal with professional requirements and stressors; alternatively, physicians might not work anymore in professional patient care. Moreover, the high level professional role associated with social and financial benefit might be a protective factor against burnout (Cheli et al. 2021).

The work setting of physicians may have a strong impact on professional satisfaction: In contrast to data from the US with higher burnout rates among physicians working in their own private practices than those working in academic medical centers or other practices (Dyrbye et al. 2011, 2013), this survey showed a lower prevalence of exhaustion in self-employed physicians. Given that healthcare systems in Germany are different from those in the US, it can be assumed that physicians in outpatient oncological practices are less confronted with stress factors, such as rotating-shift work or night work. Furthermore, self-employment might allow a better control of the workload leading to less stress and a higher professional satisfaction (Williams et al. 2002). Furthermore, in our cohort, oncologists in outpatient practices tended to be older; thus, the reduced rate of exhaustion might also be attributed to the higher age and the higher grade of experience. Physicians’ psychological burden has a strong impact on medical care. In a comprehensive systematic review in 2016, Hall et al. (2016) have reported a high level of evidence for relations between self-reported medical errors and psychological burden. A possible explanation for this relationship is the emergence of cognitive limitations. These findings stress the importance of physicians’ health to ensure quality patient care.

Though the rate of exhaustion was high in this survey, the rates of disengagement were lower than expected with a mean value of 1.65 and less than 10% showing a high rate of disengagement. This prevalence is lower than the prevalence of disengagement of physicians in other disciplines, such as emergency medicine (Chernoff et al. 2019) or medical staff in general. These findings imply high demands of physicians to fulfill job requirements despite imminent or actual overburdening in daily work.

### Strengths and limitations

This study is the first to examine two dimensions of burnout in a German sample of oncologists. We had the opportunity to reach several oncologists through the cooperation with the AIO. The prevalence of physicians additionally board certified in palliative care is higher (exceeding 20–30%) among oncologists than in other medical disciplines (5% among all medical disciplines in Germany) (KV 2020). This is mainly caused by the fact that patients treated in a palliative setting represent a substantial part of all patients treated in an in- or outpatient setting. As a consequence, aspects of palliative and supportive care are integral part of the daily work among oncologists.

However, this survey has several limitations. First, the number of physicians that responded was limited; physicians with a higher symptom load might have reported at a higher frequency, leading to an overestimation of the prevalence of burnout, though the prevalence of burnout reported here is in line with data published.

Second, since the participants were members of a professional society, the work demands, and job characteristics might not reflect clinical routine in Germany. Third, since this survey was conducted in summer 2020 after the first peak of the coronavirus disease 2019 (COVID-19) pandemic, additional stressors associated with this might have influenced the results of this survey. In contrast, during the survey running, the incidence rates of COVID-19 were low in Germany with only few major structural issues occurring in hospitals. The main limitation of this study is related to its cross-sectional design, which does not allow either temporal or causal inferences. Further investigations with consecutive measurements are required to obtain a more detailed understanding of burnout among oncologists.

## Conclusion

This survey highlights a high rate of exhaustion among German oncologists, with a focus on professional burden during the early years of career. More support is necessary to mitigate potential stressors for medical personnel within the healthcare system. To ensure high-quality medical care, physicians should be offered preventive mental healthcare services early in their careers.

## Data Availability

The datasets generated and/or analyzed during this study are available from the corresponding author on reasonable request.
